# To what extent do cataracts and cataract surgery change perception?

**DOI:** 10.1167/jov.25.10.13

**Published:** 2025-08-27

**Authors:** Simona Garobbio, Hanna Zuche, Ursula Hall, Nina L. Giudici, Chrysoula Gabrani, Hendrik P. N. Scholl, Michael H. Herzog

**Affiliations:** 1 Laboratory of Psychophysics, Brain Mind Institute, École Polytechnique Fédérale de Lausanne (EPFL), Lausanne, Switzerland; 2 Department of Ophthalmology, University Hospital Basel, Basel, Switzerland; 3 Institute of Molecular and Clinical Ophthalmology Basel, Basel, Switzerland; 4 Department of Clinical Pharmacology, Medical University of Vienna, Vienna, Austria; 5 Pallas Kliniken AG, Pallas Klinik Zürich, Zürich, Switzerland; 6 European Vision Institute, Basel, Switzerland

**Keywords:** cataracts, cataract surgery, visual function tests, vision vs. cognition

## Abstract

Cataract surgery is the most commonly performed surgical procedure worldwide and is typically associated with an improvement in visual acuity (VA). This study aimed to examine how various visual functions, beyond VA and contrast sensitivity, are affected by cataracts and how they change after cataract surgery. We assessed 28 adults (aged 55–85 years) with vision-impairing cataracts using a comprehensive battery of visual tests at four visits: before surgery, 1 week after surgery of the first eye, 1 week after surgery of the second eye, and 1 month after the second surgery. Tests included VA, contrast sensitivity, coherent motion (CMot), orientation discrimination, visual search, and reaction time, assessed monocularly and binocularly. Both a cognitive and a self-assessment questionnaire were administered at the first and last visits. Results indicated that cataracts impaired all visual functions except CMot. Postoperatively, VA, contrast sensitivity, and CMot improved significantly, with marginal gains in orientation discrimination and no change in visual search or reaction times. Improvements were greater after the first surgery. Also, stronger correlations between low-level visual functions, cataract severity, and self-assessment scores were observed for the first operated eye. Cognitive scores correlated significantly with performances in CMot, orientation discrimination, and visual search. These findings suggest that cataracts strongly affect low-level visual processing, whereas higher-level tasks may be maintained through cognitive compensation. Cataract surgery recovers performance in most but not all visual tests, highlighting the importance of considering visual function beyond VA, as well as cognitive functioning, in ophthalmic clinical care.

## Introduction

Cataracts are a leading global cause of blindness in those aged 50 years and older as of 2020, with an estimated 16 million people worldwide (Asbell et al., 2005; Bourne et al., 2021). In total, 78.8 million people worldwide experience moderate to severe visual impairment owing to cataracts (Bourne et al., 2021). Cataracts are defined as an opacification of the natural intraocular lens, which, if left untreated, eventually leads to significant vision loss (Asbell et al., 2005). Cataracts develop slowly and painlessly, meaning that vision and lifestyle can be affected without the patient realizing it. Cataract surgery, which is by far the most prevalent surgical procedure among all medical disciplines (Rossi et al., 2021), involves the removal of the opaque lens and implantation of an artificial intraocular lens. It is one of the most cost-effective interventions in health care for improving visual impairment (Burton et al., 2021). Despite the advantages of modern cataract surgery, it remains unclear how exactly visual performance changes after surgery. The implanted lens differs significantly in several ways from the biological lens. Over the past two decades, new diagnostic tools have revolutionized ophthalmic care. With optical coherence tomography (OCT), high-resolution images of the human eye and retina can be obtained in vivo (Schuman et al., 1993; Yaqoob, Wu, & Yang, 2005). However, despite this enhanced capability for imaging structural abnormalities, little is known about their effect on visual perception.

Research in visual perception has also undergone dramatic changes in the last decade. Using batteries of perceptual tests, studies have shown that perceptual functions are largely independent, meaning that there are few or only marginal correlations between perceptual tests (e.g., Bosten et al., 2017; Bosten & Mollon, 2010; Cappe, Clarke, Mohr, & Herzog, 2014; Garobbio, Kunchulia, & Herzog, 2024; Shaqiri et al., 2019; for reviews see Bosten, Mollon, Peterzell, & Webster, 2017; Peterzell & Kennedy, 2016; Tulver, 2019). For instance, Cappe et al. (2014) tested 40 participants with 6 basic visual tests (e.g., vernier acuity, visual acuity [VA], and contrast sensitivity), and reported weak correlations between pairs of tests. Similarly, Shaqiri et al. (2019) assessed performance in 19 visual paradigms, testing basic (e.g., VA and contrast sensitivity) to more complex (e.g., visual search [VSrch] and motion perception) visual functions in 104 young and 92 older participants. Between-task correlations in both groups were very low, even though test–retest reliability was good. This finding suggests that poor performance in one test, such as VA, does not predict poor performance in another, such as orientation discrimination (Ori).

Because there are only weak correlations, the question arises how visual functions, other than VA and contrast sensitivity, are affected by cataracts and how they change after cataract surgery (Liu et al., 2017; Shandiz et al., 2011). Because the visual system is highly heterogeneous, relying on a single test, as is common in vision and ophthalmological studies, is no longer sufficient to capture its complexity. Instead, batteries of tests are necessary to assess the diverse components of visual processing. In this line, our study does not necessarily aim for a single finding, but rather a set of interconnected results. This multifaceted approach is intentional and essential, as it reflects the multidimensional nature of both the visual system and its diseases.

Therefore, to identify and characterize visual deficits caused by cataracts and the improvements after cataract surgery, patients performed a battery of six visual tests targeting several stages of the visual processing. Most of the tests were performed binocularly, because binocular vision reflects normal viewing. In addition, monocular tests were conducted to specifically examine the changes after the first eye was operated, with the other eye still suffering from cataract. Together, this dual approach of binocular and monocular testing provides a comprehensive picture of how cataracts impact vision before and after surgery. Furthermore, recent studies have shown an association between vision impairments and cognitive impairments; however, this relationship may not be causal, because age could act as a confounding variable influencing both vision and cognitive decline (Arsiwala et al., 2021; Garobbio, Pilz, Kunchulia, & Herzog, 2022; Mine et al., 2016; Tran et al., 2020; Varadaraj et al., 2021; Zheng et al., 2018; for reviews see Shang, Zhu, Wang, Ha, & He, 2021; Vu et al., 2021). By including patient-reported outcome measures such as the Visual Function Index (VF-14) questionnaire, as well as the Montreal Cognitive Assessment (MoCA) for cognitive function, this study offers a unique opportunity to test whether changes in visual performance on various visual function tests influence cognitive functioning.

## Methods and materials

### Participants and general procedure

Twenty-eight patients aged 55 to 85 years (mean, 72.3 ± 7.6 years) were recruited at the Department of Ophthalmology, University Hospital Basel, and enrolled into the study. Inclusion criteria included vision-impairing binocular cataracts, age older than 50 years, otherwise clear optical media as assessed with a slit lamp, sufficient pupillary dilation for adequate imaging and function tests, and a score of at least 16 on the MoCA test. Exclusion criteria included additional coexistent ocular disease that could affect visual function or ocular morphology, and VA result (as measured with Nidek Autorefractor Oculus/Nidek AR) higher than 1.3 logarithm of the minimum angle of resolution (20/400 Snellen equivalent). The eye with worse objective VA or, if both had similar VA, worse subjective vision, was operated on first. The second eye was operated 2 to 4 weeks after the surgery on the first eye. All patients received monofocal aspheric intraocular lenses and no premium intraocular lens (multifocal or enhanced-depth of vision intraocular lens) was implanted.

Patients attended four visits: 2 weeks before surgery (visit 1), 1 week after surgery on the first eye (visit 2), 1 week after surgery on the second eye (visit 3), and 6 weeks after the second surgery (visit 4). At visit 1, patients underwent a medical history assessment, demographic information was collected, and cataract severity was determined using the Simple Pre-Operative Nuclear Classification Score (SPONCS) grading. At each visit, patients performed six visual tests: quick contrast sensitivity function (qCSF), Freiburg VA, Ori, coherent motion (CMot), reaction time (RT), and VSrch. At the first and last visit, participants also completed the MoCA test (Nasreddine et al., 2005), filled out the VF-14 questionnaire (Chiang, Fenwick, Marella, Finger, & Lamoureux, 2011), and underwent spectral-domain OCT (Spectralis OCT; Heidelberg Engineering, Germany, Settings: 193 horizontal raster scans covering 20° × 20°, automated real-time averaging at 16 frames, tracking function activated, “high-speed” setting) imaging of the macula. In the spectral-domain OCT scans, the central subfield thickness was measured to exclude cystoid macular edema, which could have influenced the results.

The study adhered to the Declaration of Helsinki and was approved by the Ethics Committee of the Canton of Basel in Switzerland (approval number: 2022-01873). All participants provided written informed consent before the experiment, were informed that they could withdraw from the experiment at any time, and were reimbursed for their participation.

Patient demographics for each visit are provided in Table 1.

**Table 1.** Description of the 28 patients’ demographics across the four visits. *Notes*: 1OE = first operated eye; 2OE = second operated eye. For continuous variable, the mean and standard deviation is provided; logMAR = logarithm of the minimum angle of resolution.

	Visit 1	Visit 2	Visit 3	Visit 4
Gender (F/M)	11/17			
Age	72.29 ± 7.62			
Education (n)				
Secondary school	1			
High school	14			
University	12			
Race (White/others)	28/0			
BMI	25.45 ± 3.62			
Smoking years	19.43 ± 21.89			
Smoker (no/past/now)	11/13/4			
1OE (right/left)	15/13			
MoCA	25.47 ± 2.91			25.68 ± 3.37
VF-14	82.04 ± 14.5			86.04 ± 12.92
Slit lamp 1OE^a^ (normal/abnormal)	25/3	25/3	25/3	25/3
Slit lamp 2OE^a^ (normal/abnormal)	26/2	26/2	26/2	26/2
VA (logMAR) 1OE^b^	0.26 ± 0.20	0.05 ± 0.11	0.03 ± 0.12	0.004 ± 0.132
VA (logMAR) 2OE^b^	0.16 ± 0.13	0.11 ± 0.12	0.004 ± 0.10	−0.032 ± 0.10
Tension 1OE^c^	15.0 ± 2.97	13.96 ± 3.86		12.75 ± 2.78
Tension 2OE^c^	15.54 ± 2.90		14.38 ± 3.65	13.36 ± 3.11
Axial length 1OE	23.88 ± 1.51			
Axial length 2OE	23.86 ± 1.41			
OCT_CST 1OE^d^	244.54 ± 26.30			259.46 ± 31.63
OCT_CST 2OE^d^	240.18 ± 24.19			258.54 ± 30.75
SPONCS grading 1OE	3.07 ± 0.65			
SPONCS grading 2OE	2.91 ± 0.67			
Medications (%)				
Antihypertensive	34.5%			
Antihyperlipidemic	24.1%			
Eye medications	10.3%			
Antidiabetic	17.2%			
Sedatives	6.9%			
Other^e^	75.9%			

a Abnormal slit lamp findings included to iris defects in one patient, very mild cornea guttata in another, and map–dot–fingerprint–dystrophy in a third patient. These findings were not visually impairing.

b Autorefraction (NIDEK AR-1) was performed to measure each patient's refractive error, allowing the provision of trial glasses with the correct refraction for use while performing the functional visual tests.

c Tension was always normal, indicating that none of the patients had vision-disturbing glaucoma.

d OCT central subfield thickness (OCT_CST) increased significantly, as measured by the Wilcoxon test, between the first and last visits for both the first operated eye (W = 65; *p* = 0.003) and the second operated eye (W = 55.5; *p* = 0.001). This increase may indicate mild postoperative inflammation, as expected. No cystoid macular edema (with intraretinal fluid) was present on OCT in any patient.

e None of these medications are known to affect vision or cognition.

### Tests and extracted variables

#### Functional visual tests

Participants wore trial glasses with measured refraction values, adjusted by +0.5 diopters for 2 m testing because of accommodation before surgery. After surgery this step was omitted. During monocular tests, the untested eye was covered with a frosted lens.

##### qCSF

**Figure 1.**
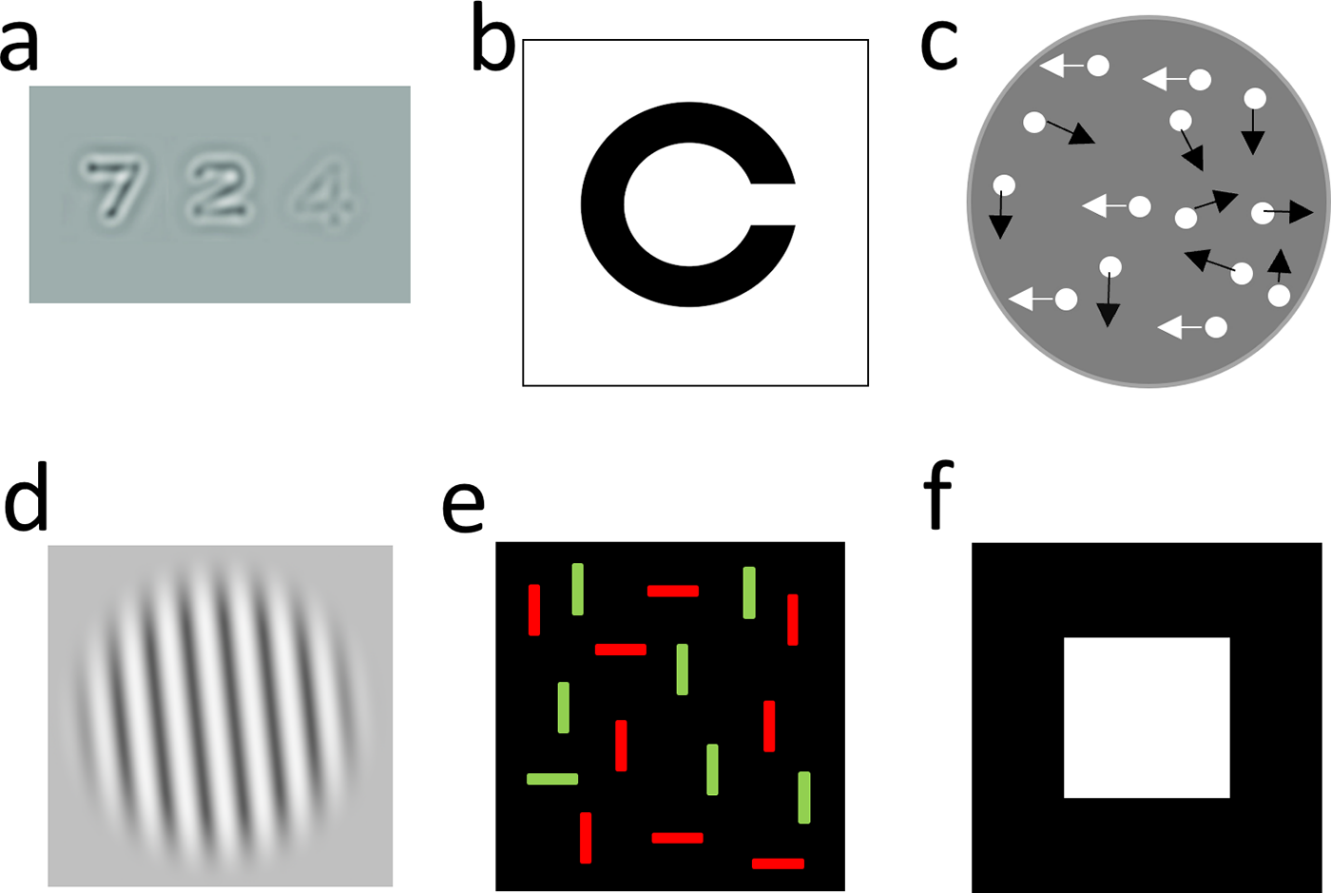
Illustrations of the tests used. (**a**) qCSF test. (**b**) Freiburg VA. (**c**) CMot (only a subset of dots is shown, with arrows indicating motion directions, though arrows were not present in the actual test). (**d**) Ori test. (**e**) VSrch test. (**f**) RT test.

qCSF testing was developed by Lesmes, Lu, Baek, and Albright (2010). The test was conducted in a room with standardized illumination of approximately 7 lux, using a large LED screen (1,920 × 1,080 pixels, NEC MultiSyncP404 LDC Monitor) from a manifold contrast vision meter (Adaptive Sensory Technology, San Diego, CA). Participants sat 3 m from the screen. The stimuli were filtered Sloan letters spanning 19 possible spatial frequencies (1.19–30.95 cycles per degree) and 128 possible contrasts at a luminance of 95.4 cd/m^2^ (Figure 1a). Participants were asked to read the optotypes presented, and their responses were recorded (as correct, incorrect, or optotype not seen) on a tablet computer by a trained study nurse. The following metrics were exported from the qCSF platform: contrast acuity (qCA) in logCPD (defined as the *x* axis intersection point with the lowest contrast sensitivity and the highest resolvable spatial frequency of the CSF curve), contrast sensitivity values at spatial frequencies of 1, 1.5, 3, 6, 12, and 18 CPDs, and the area underneath the logarithmic contrast sensitivity function (aulcsf) in logCS. The test was conducted monocularly, once for each eye.

##### VA, CMot, Ori, VSrch, and RT

Stimuli of these five tests were displayed on an LCD ASUS VG27AQ monitor (59.6 cm × 33.5 cm, 2,560 × 1,440 pixels, 144 Hz). Participants sat 2 m from the screen in a dimly illuminated room. Unless otherwise noted, responses were provided using hand-held push buttons. An auditory feedback tone was provided after incorrect responses in VA, CMot, and Ori tests to enhance response consistency through faster learning, better motivation, and minimized motor bias. Practice trials (18 for VA, VSrch, and RT, 24 for Ori, and 32 for CMot) were provided before testing to ensure proper understanding of the tests. After practice, tests were presented in the following order: binocular testing of all five tests, monocular testing with the first operated eye of Ori, and monocular testing with the second operated eye of CMot. This sequence was repeated to assess test–retest reliability. Please note that, owing to time limitations, we prioritized binocular testing over monocular testing, because binocular vision reflects normal viewing conditions. Moreover, monocular performance usually correlates between the two eyes and with binocular conditions (Cretenoud, Grzeczkowski, Kunchulia, & Herzog, 2021). VA and RT had 40 trials, and all other tests had 60 trials. For tests measuring thresholds (all except RT and VSrch), test levels were determined using the QUEST adaptive procedure (Watson & Pelli, 1983), aiming for a 75% correct response rate, except for VA, where the threshold corresponded to 62.5% owing to the four-alternative forced-choice format. We discarded 2.5% of all blocks because the estimated threshold fell outside the range of tested values. Stimulus programs were implemented using the Psychophysics Toolbox (Brainard, 1997) and GNU Octave (Eaton, Bateman, Hauberg, & Wehbring, 2021). Descriptions of each test are as follows.

VA: VA was measured using the Freiburg VA test (Bach, 1996). Landolt-C black optotypes with randomized gap orientations were presented on a white background with 100 cd/m^2^ luminance (Figure 1b). Participants indicated the gap direction (“up,” “down,” “left,” or “right”) by pressing the corresponding button on a keypad. Each optotype was presented until the participant responded, and optotype size changed adaptively across trials. The test provided a decimal VA score.

CMot: Participants discriminated between leftward and rightward motion in a random dot kinematogram, where a specified ratio of dots moved coherently in one direction, and the remaining dots moved randomly. A total of 1,000 dots (dot diameter: 4 arcmin; luminance: 100 cd/m^2^) moved at 2.86 arcdeg/s within a circular area (diameter: 8 arcdeg; background luminance: 10 cd/m^2^) for 400 ms (Figure 1c). The metric of interest was the ratio of coherently moving dots.

Ori: This task was based on a paradigm by Tibber, Guedes, and Shepherd (2006). Participants identified whether an elongated Gabor patch, with an orientation that changed adaptively, was oriented clockwise or counterclockwise relative to the vertical axis. The Gabor patch had a Michelson contrast of 80%, mean luminance of 40 cd/m^2^, spatial frequency of 3.3 cycles/arcdeg, an envelope sigma of 0.57 arcdeg along and 0.19 arcdeg perpendicular to its orientation, and a presentation duration of 200 ms (Figure 1d). The measure of interest was the orientation threshold in degrees.

VSrch: Based on Theeuwes and Kooi (1994), this test required participants to search for a green horizontal line within an array of distractors (green vertical and red horizontal lines; Figure 1e). Sixteen lines (length: 1,600 arcsec; width: 450 arcsec) were displayed in a square area (side length: 6 arcdeg) until response. Participants indicated whether or not the display contained a green horizontal line, which was present in 50% of the trials. Average RT for correct responses and percentage correct were combined into an inverse efficiency score (i.e., RT for correct responses/percentage correct) (Vandierendonck, 2018).

RT: The RT test was based on the Hick paradigm (Hick, 1952). Participants pressed a push button immediately after a white square (size: 3 arcdeg; duration: 5 s; luminance: 100 cd/m^2^) appeared on a black background (Figure 1f). The intertrial interval varied randomly between 1.5 and 3.5 s to prevent anticipation.

#### Cataract grading (SPONCS)

Cataracts were initially graded using the internationally approved Lens Opacities Classification System (LOCS III; Chylack et al., 1993), which evaluates the severity of nuclear opalescence, cortical opalescence, and posterior subcapsular involvement on a scale from 1 to 3 (1–4 for cortical opalescence). These LOCS III grades were then converted into the SPONCS (Mandelblum et al., 2020). The conversion from LOCS III to SPONCS was as follows: for nuclear opalescence, a grade 1 (mild) on the LOCS III scale corresponds with a SPONCS score of 2, grade 2 (moderate) corresponds with a score of 3, and grade 3 (severe) corresponds with a score of 4. Additionally, if there was any involvement of the cortical or posterior subcapsular regions, 0.5 points were added to the SPONCS score. No patient showed +3 cortical or +3 posterior subcapsular opalescence, so the 0.5 points added were reasonable in our cohort. This conversion process provides a continuous scale, which is better suited for statistical analysis.

#### Questionnaires

##### MoCA

The MoCA was developed by Nasreddine et al. (2005) and is widely used as a screening tool for mild cognitive impairments. This 10-minute paper-and-pencil task assesses visuospatial skills, executive functions, language, memory, attention, calculation, abstraction, and orientation. A maximum score of 30 can be achieved. A score of 15 or less indicates dementia, a score between 16 and 25 indicates mild cognitive impairment, and a score of 26 or higher indicates healthy participants. The MoCA has been validated in various populations and countries (Ciesielska et al., 2016; Freitas, Simões, Marôco, Alves, & Santana, 2012; Julayanont & Nasreddine, 2017). Participants performed the MoCA in their mother tongue. To prevent participants from remembering the test, version 7 was used at visit 1, and alternative version 2 was used at visit 4 (Costa et al., 2012). No participant scored less than 19 points.

##### VF-14

The VF-14 questionnaire is a brief tool designed to measure functional impairment owing to cataracts (Chiang et al., 2011; Steinberg et al., 1994). It contains 14 questions about vision-dependent activities performed in everyday life, such as reading, recognizing people, seeing steps, doing fine handiwork, writing, playing games, doing sports, cooking and preparing meals, watching television, and driving. The difficulty of performing each activity is rated on a five-category Likert scale: (0) not possible, (1) a lot of difficulty, (2) some difficulty, (3) a little difficulty, and (4) no difficulty at all. Scores are calculated by summing the responses and dividing by the number of valid responses. The result is then multiplied by 25 to yield a final score ranging between 0 and 100, with 0 representing the worst possible functioning and 100 representing the best possible functioning.

### Test–retest reliability

To detect participants who showed large performance differences across the two repetitions of the functional tests, we merged the data from the four visits, calculated the absolute score differences, and identified outliers using a 3.5-standard deviation criterion (Iglewicz & Hoaglin, 1993). This led to the exclusion of 0.03% (23 test results out of 784; i.e., of 28 participants × 7 tests × 4 visits; see Supplementary Table S1 in the Supplementary Material).

Before averaging the scores of the two repetitions to obtain a single score for each participant for each test, we assessed test–retest reliability by computing two-way mixed effects models (i.e., intraclass correlation of type [3,1] or ICC31; Koo & Li, 2016; Shrout & Fleiss, 1979). We also visually inspected the participants’ scores across the two test repetitions using scatter plots (see Supplementary Table S2 and Supplementary Figure S1 in the Supplementary Material). ICCs and scatter plots indicated significant test–retest reliability for all tests, with large effect sizes (ICCs for binocular tests: VA 0.95, CMot 0.65, Ori 0.79, VSrch 0.95, RT 0.86; ICC for monocular tests: Ori with first operated eye 0.78, CMot with second operated eye 0.76).

From this point forward, the average scores from the two test repetitions were used.

### Data analysis

Approximately one-half of the variables’ distributions violated the normality assumption as assessed by the Shapiro-Wilk test, mainly owing to skewness (Supplementary Figures S2, S3, and S4 in the Supplementary Material). Therefore, nonparametric statistical tests were used.

Participants with missing scores were not excluded, and no data imputation was performed. Instead, pairwise deletion was applied to compute correlations and *t* tests. Please note that a higher score indicates better performance in all tests, except for CMot, Ori, VSrch, and RT.

#### Healthy controls vs. cataract patients before surgery

To assess the extent of patients’ deficits at baseline (i.e., visit 1), we compared their performance with data from 28 healthy controls, aged 59 to 81 years (mean, 68.4 ± 7.2 years). These controls were a subset of 49 older adults who participated in a parallel study at the Department of Ophthalmology, University Hospital Basel (MuMoVi study; data not yet published). From the original 49 participants, we excluded four who had cataract surgery, eight with mild cataracts, two who did not perform the qCSF test, and the seven youngest participants to match the size of the control group to that of the patient group, resulting in 28 controls. The controls performed the following tests monocularly with their nondominant eye: qCSF, VA, Ori, CMot, and VSrch. The procedure and setup were identical to those used in our study.

A comparison of demographic variables between patients and controls is reported in Supplementary Table S3.

To test whether test performance differed between the two groups, we conducted a Mann–Whitney *U* test. Bonferroni–Holm correction (Holm, 1979) for multiple comparisons was applied across the tests performed binocularly, with the first operated eye, or with the second operated eye of the patients.

Effect sizes were interpreted using Cohen's guidelines (1988), with effect sizes of 0.2, 0.5, and 0.8 considered small, medium, and large, respectively. Effect sizes provide a standardized measure of the magnitude of the effect, and are calculated as the ratio between the difference in means of two groups (or two visits) and the pooled standard deviation. Thus, if the standard deviation is small, even a small mean difference could be meaningful, whereas if the standard deviation is large, a bigger mean difference is needed to show an effect. For instance, an effect size of 0.5 indicates that the difference between the two groups is one-half the size of the standard deviation, representing a moderate effect, whereas an effect size of 1.5 means that the difference between the two groups is 1.5 times the size of the standard deviation, indicating a large effect. The sign of the effect size indicates the direction of the effect: a positive effect size suggests that the mean of the first group (or time point) is higher than the second, whereas a negative effect size indicates that the second group (or time point) has a higher mean.

#### Performances across visits

To visualize performance across visits, we plotted the individual scores for each test at each visit. To assess whether performance changed across visits, we conducted a linear mixed-effect model (LMM) for each variable. We confirmed that the residuals and random effects followed a normal distribution, thus meeting the assumptions required for using the LMM. When the LMM identified a significant effect of the visit, we performed Wilcoxon signed-rank tests for pairwise comparisons between visits. The resulting *p* values were adjusted for multiple comparisons using the Benjamini–Hochberg false discovery rate correction (Benjamini & Hochberg, 1995) across the tests performed binocularly, with the first operated eye or with the second operated eye. Given the large number of comparisons, we opted for false discovery rate correction rather than Bonferroni–Holm correction, because it offers a better balance between controlling false positives and maintaining statistical power. In addition, the use of a false discovery rate is justified, because we are interested in making statements based on the overall pattern of results rather than relying on isolated significant findings.

#### Performance vs. questionnaires and SPONCS

We tested for potential associations between test performances and the scores in MoCA and VF-14 by computing Spearman's correlations, and between test performances and SPONCS by computing Kendall's correlations. Kendall's correlations were used because SPONCS is an ordinal scale with fewer than 10 levels. To interpret the strength of correlations, we used Cohen's guidelines (1988), which consider correlation coefficients of 0.1, 0.3, and 0.5 as small, medium, and large in magnitude, respectively.

## Results

The mean and standard error for each test at each visit are provided in Supplementary Table S4 in the Supplementary Material. Results for the control group are reported in Supplementary Table S5 in the Supplementary Material.

### Healthy controls vs. cataract patients before surgery

**Figure 2.**
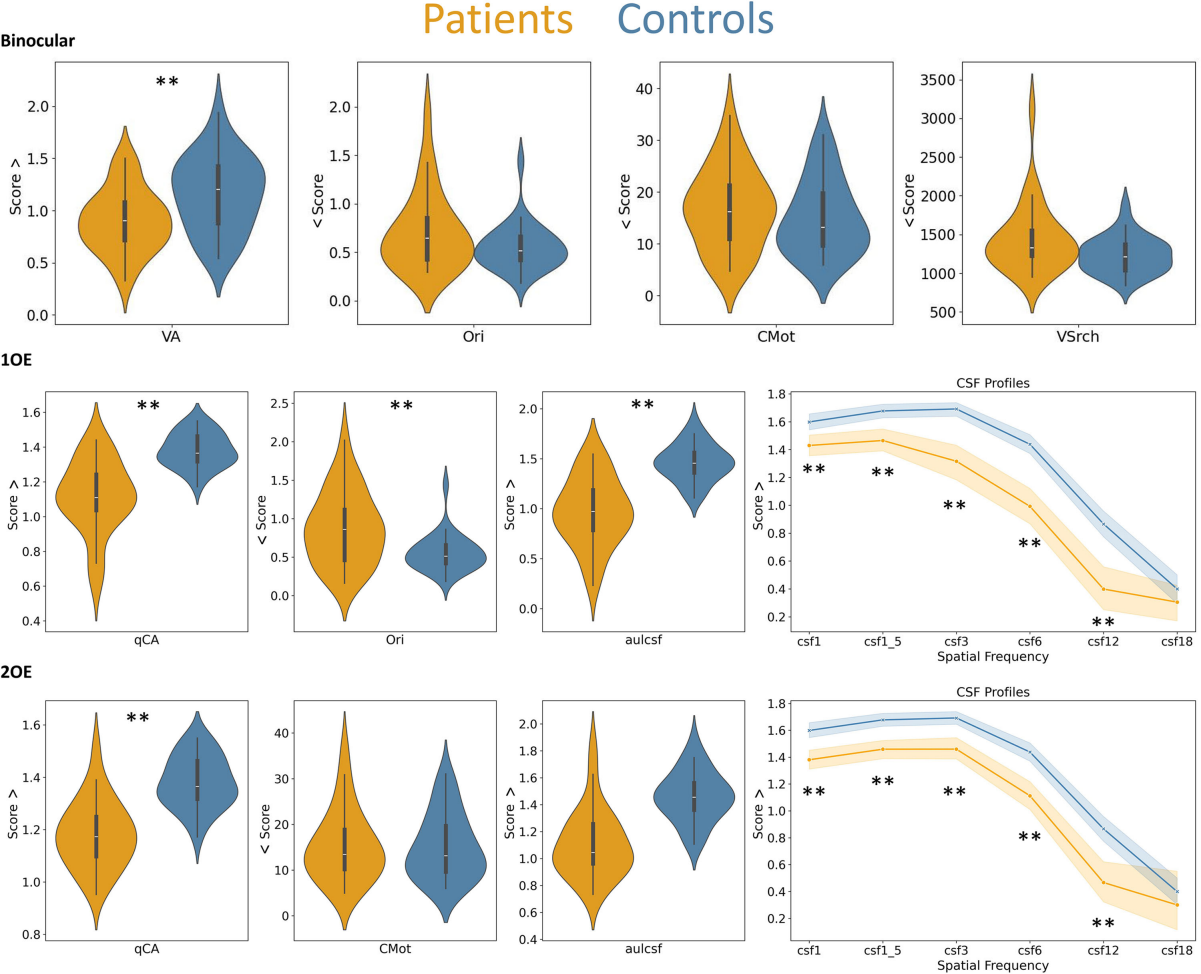
Visualization of the results of the visual function tests for patients (orange) and controls (blue) at baseline (before cataract surgery). The first row shows the violin plots of the tests performed binocularly by patients vs. controls. The second row shows violin plots and line plots for the CSF variables of the tests performed with the first operated eye (1OE) for patients vs. controls. The third row shows violin plots and line plots for the CSF variables of the tests performed with the second operated eye (2OE) for patients vs. controls. Higher scores (Score >) indicate better performance in all tests except for Ori, CMot, and Vsrch (< Score). Asterisks (**) indicate significant results after Bonferroni–Holm correction for multiple comparisons (*p* < 0.05). Details from the statistical tests are reported in Supplementary Table S6 in the Supplementary Material. aulcsf = area under the logarithmic contrast sensitivity function; csf1–csf18 = contrast sensitivity values of the spatial frequencies at 1–18 CPDs, respectively; qCA = contrast acuity; VA = Freiburg VA.

Figure 2 illustrates the comparison between cataract patients at first visit (i.e., before cataract surgery) and healthy controls. The control group performed tests monocularly with their nondominant eye, and the patient group performed the tests either binocularly (first row), monocularly with their first to be operated eye (first operated eye, second row), or monocularly with their second to be operated eye (second operated eye, third row). Results from statistical tests are reported in Supplementary Table S6 in the Supplementary Material.

Patients showed statistically significantly impairment, particularly in VA and contrast sensitivity variables, as well as when the binocular VA of patients was compared with the monocular VA of controls. For all VA and contrast sensitivity variables, except for csf18, the effect sizes were large and comparable between the first and the second operated eyes (Cohen's d ranging from −1.16 to −2.54). Please note that the lack of a difference for csf18 is likely due to the small sample size of patients (i.e., 16 of 28 patients were able to complete the test; see Supplementary Table S4 in the Supplementary Material).

A significant decrease in patients’ performance was also observed for the Ori test when monocular performance was compared across the two groups, but not when patients performed the test binocularly, even though the effect size was moderate (d = 0.41). A large effect size was also observed in the VSrch test, with patients performing worse than controls (d = 0.60). Only in the CMot was there no evidence of deficient performance in patients.

### Performance across visits

**Figure 3.**
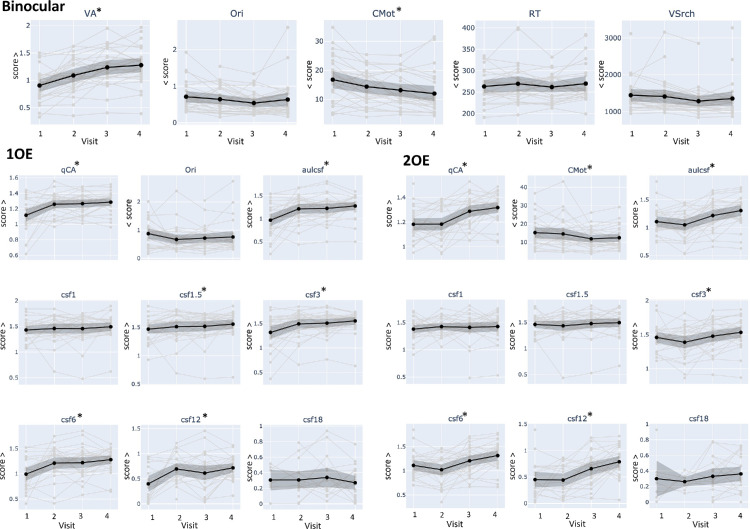
Traces of individual trajectories for binocular tests (top), tests performed with the first operated eye (1OE), and tests performed with the second operated eye (2OE) at each visit. The black line depicts the average trajectory and 95% confidence intervals. Asterixis (*) indicate a significant effect of visit as determined by the LMM. Detailed statistical results from the LMM are provided in Supplementary Table S7 in the Supplementary Material, and results from the Wilcoxon signed-rank tests for pairwise comparisons between visits are reported in Table 2. A higher score indicates better performance in all tests (score >) except for VSrch, RT, CMot, and Ori (<score).

Figure 3 shows the individual traces across the four visits in the background, with averages and 95% confidence intervals highlighted in black, and Table 2^3^ reports the statistical results for pairwise comparisons between visits for tests where the LMM indicated a significant effect of visit (see LMM results in Supplementary Table S7 in the Supplementary Material).

**Table 2.** Results from Wilcoxon signed-rank tests for pairwise comparisons between visits (1, 2, 3, and 4) for tests that showed a significant effect of visit as determined by the LMM.

	1 vs. 2			1 vs. 3			1 vs. 4			2 vs. 3			2 vs. 4			3 vs. 4		
	*W*	*pBH*	*d*	*W*	*pBH*	*d*	*W*	*pBH*	*d*	*W*	*pBH*	*d*	*W*	*pBH*	*d*	*W*	*pBH*	*d*
Binocular																		
VA	59	**0.003**	−1.90	23	0.002	−3.67	20	**1.9e−4**	−4.17	94	**0.048**	−1.05	53	**0.002**	−2.07	148	0.331	−0.44
CMot	72	**0.014**	1.46	32	**8.4e−4**	2.84	32	**8.4e−4**	2.98	131	0.264	0.52	82	**0.014**	1.37	84	**0.014**	1.34
First operated eye																		
qCA	51	**0.006**	−1.87	44	**0.006**	−2.0	46	**0.006**	−2.06	166	0.589	−0.25	129	0.364	−0.55	161	0.414	−0.42
aulcsf	40	**0.002**	−2.30	40	**0.002**	−2.27	43	**0.002**	−2.30	175	0.539	−0.27	147	0.252	−0.57	135	0.252	−0.60
csf1.5	94	0.208	−0.92	108	0.293	−0.71	97	0.208	−1.00	202	0.991	−0.01	172	0.585	−0.31	152	0.382	−0.51
csf3	57	**0.009**	−1.71	57	**0.009**	−1.82	49	**0.008**	−2.08	176	0.546	−0.27	152	0.323	−0.52	154	0.323	−0.50
csf6	36	**0.007**	−2.05	31	**0.003**	−2.46	34	**0.003**	−2.29	180	0.616	−0.22	136	0.199	−0.69	150	0.278	−0.54
csf12	12	**0.021**	−2.67	34	**0.047**	−1.51	25	**0.021**	−2.00	125	0.975	0.02	113	0.815	−0.22	119	0.378	−0.56
Second operated eye																		
qCA	129	0.381	−0.42	45	**0.001**	−2.22	40	**0.001**	−2.43	40	**0.001**	−2.43	40	**0.001**	−2.56	144	0.342	−0.49
CMot	153	0.279	0.51	76	**0.022**	1.60	85	**0.022**	1.43	121	0.127	0.88	131	0.155	0.76	155	0.279	−0.49
aulcsf	162	1.00	−0.01	90	**0.037**	−1.1	46	**0.005**	−1.06	70	**0.015**	−1.50	42	**0.002**	−2.48	103	**0.036**	−1.12
csf3	147	0.692	0.19	153	0.692	−0.25	91	0.100	−1.09	107	0.100	−0.96	66	**0.035**	−1.58	125	0.116	−0.83
csf6	123	0.449	0.36	116	0.161	−0.72	67	**0.019**	−1.56	87	**0.029**	−1.28	47	**0.004**	−2.28	114	0.066	−0.98
csf12	67	0.979	−0.03	31	**0.016**	−1.82	32	**0.015**	−1.93	36	**0.016**	−1.74	24	**0.009**	−2.42	90	0.062	−1.00

The *pBH* values were corrected for multiple comparisons with Bonferroni–Holm within each test (i.e., across the six pairwise visit comparisons). A high score indicates better performance in all tests except for Ori, CMot, RT, and VSrch. Therefore, a negative effect size indicates performance improvement in all tests except for Ori, CMot, RT and VSrch, where performance improvement is indicated by a positive effect size. Significant result (*p* < 0.05) are shown in bold.

**Table 3.** Correlations (*r, p*) between visual tests scores and MoCA, VF-14, and SPONCS. *Notes*: 1OE = first operated eye; 2OE = second operated eye).

	SPONCS 1OE	SPONCS 2OE	VF-14 visit 1	VF-14 visit 4	MoCA visit 1	MoCA visit 4	MoCA controls
Binocular tests							
VA	−0.02, 0.87	−0.01, 0.97	0.23, 0.25	0.04, 0.85	0.11, 0.58	0.16, 0.42	−0.12, 0.56
Ori	−0.03, 0.84	0.15, 0.33	0.06, 0.79	−0.13, 0.50	−**0.53, 0.01**	−**0.32, 0.09**	
CMot	−0.14, 0.35	−0.12, 0.41	0.02, 0.93	−0.09, 0.64	−**0.38, 0.05**	−**0.54, 0.0**	
RT	−0.11, 0.49	−0.22, 0.17	0.02, 0.92	0.10, 0.63	−0.08, 0.7	−**0.4, 0.04**	
VSrch	0.08, 0.6	−0.23, 0.13	−0.11, 0.60	−0.27, 0.19	−**0.3, 0.13**	−**0.48, 0.01**	0.09, 0.65
1OE							
qCA	−**0.3, 0.05**		**0.39, 0.05**	−0.05, 0.82	−**0.39, 0.05**	0.23, 0.23	0.02, 0.91
Ori	0.1, 0.53		−**0.53, 0.01**	−0.03, 0.99	−**0.31, 0.11**	−0.26, 0.2	−0.19, 0.33
aulcsf	−**0.3, 0.05**		**0.5, 0.01**	−0.11, 0.57	−**0.33, 0.1**	0.22, 0.27	0.15, 0.46
csf1	−**0.35, 0.02**		0.18, 0.39	0.20, 0.30	−0.21, 0.3	−0.08, 0.69	0.09, 0.65
csf1.5	−**0.42, 0.01**		**0.34, 0.09**	0.27, 0.16	−0.21, 0.31	−0.07, 0.71	0.10, 0.63
csf3	−**0.34, 0.03**		**0.53, 0.01**	0.06, 0.75	−0.25, 0.22	0.09, 0.64	0.18, 0.36
csf6	−0.08, 0.63		**0.52, 0.01**	−0.09, 0.65	−0.22, 0.32	0.27, 0.16	0.21, 0.29
csf12	−0.21, 0.27		0.25, 0.32	−0.3, 0.15	−**0.38, 0.12**	**0.34, 0.09**	0.08, 0.68
csf18	−0.16, 0.63		**0.64, 0.12**	−0.17, 0.47	−**0.3, 0.51**	**0.34, 0.13**	0.04, 0.84
2OE							
qCA		−0.04, 0.78	0.29, 0.15	0.04, 0.83	−0.08, 0.72	0.16, 0.41	
CMot		−0.02, 0.87	−0.06, 0.75	−0.16, 0.42	−**0.39, 0.04**	−**0.46, 0.01**	−0.13, 0.51
aulcsf		−0.25, 0.11	**0.32, 0.12**	0.04, 0.83	−0.07, 0.74	0.2, 0.31	
csf1		0.06, 0.68	**0.32, 0.12**	0.11, 0.57	0.23, 0.26	**0.37, 0.05**	
csf1.5		−0.05, 0.75	0.29, 0.15	0.12, 0.54	0.28, 0.17	**0.32, 0.1**	
csf3		0.22, 0.14	0.24, 0.25	0.11, 0.58	0.08, 0.7	0.2, 0.3	
csf6		−**0.33, 0.03**	0.28, 0.17	0.02, 0.91	−0.15, 0.46	0.15, 0.46	
csf12		−0.21, 0.23	**0.53, 0.02**	−0.01, 0.98	−0.2, 0.4	0.28, 0.16	
csf18		−0.12, 0.73	−0.26, 0.62	−0.26, 0.24	0.16, 0.73	0.21, 0.35	

Pairwise deletion was applied. Effect sizes (*r*) and corresponding *p* values (*p*) are reported. Spearman correlation was used for VF-14 and MoCA, and Kandall correlation for SPONCS. Moderate to large correlation (i.e., *r* > 0.30) are indicated in bold. The significance level varies across variables owing to differences in sample sizes. Note that controls performed all tests monocularly with their nondominant eye.

VA variables (VA and qCA) showed substantial improvement for all conditions (binocular, first operated eye, and second operated eye). For contrast sensitivity, there were large improvements in aulcsf, csf3, csf6, and csf12 when measured monocularly with the first operated eye. Improvements were observed to a lesser extent for monocular testing with the second operated eye. Csf1, csf1.5, and csf18 did not show significant improvements; however, it is worth noting that more patients were able to complete csf18 at the final visit compared with baseline.

Among the tests evaluating higher-level visual functions, only CMot showed a significant improvement over visits, both binocularly and monocularly. A marginal significance was observed for Ori when performed binocularly (*p* = 0.07). No evidence of performance changes was found for RT and VSrch.

Importantly, the lack of significant differences between visit 2 and visit 3 for the tests performed with the first operated eye, and between visit 1 and visit 2 for the tests performed with the second operated eye, suggests that no learning effect occurred (because no surgery occurred between these visits for the tested eye). Interestingly, for the binocular tests, VA improved after each operation, whereas CMot showed its significant improvement after the first operation. There was almost no improvement between visit 3 and visit 4, with some exceptions for CMot binocular and aulcsf performed with the second operated eye.

### Performance vs. questionnaires and SPONCS

Between visit 1 and visit 4, there was no significant change in MoCA scores (W = 110; *p* = 0.60) and a marginal significance in VF-14 (W = 96; *p* = 0.08), indicating that patients perceive some improvements in daily visual tasks after the surgery. MoCA scores did not correlate with VF-14 (r = −0.01 at visit 1 and r = 0.05 at visit 4). MoCA scores significantly correlated with SPONCS for the first operated eye (r = 0.38; *p* = 0.01), suggesting that greater cataract severity in this eye is associated with a higher MoCA score. However, there was no significant correlation between MoCA and SPONCS for the second operated eye (r = 0.16; *p* = 0.31). VF-14 did not correlate with any SPONCS grading (r = −0.11 with first operated eye and r = −0.03 with second operated eye). Correlations between performance and SPONCS, VF-14, and MoCA are reported in Table 3 and discussed below.

#### Correlations with SPONCS

For the first operated eye, SPONCS grading correlated significantly with qCA and low spatial frequency contrast sensitivity (i.e., csf1, csf1.5, and csf3), indicating that a more severe cataract was associated with poorer performance on these tests. In contrast, SPONCS grading for the second operated eye showed a significant correlation only with csf6. No significant correlations were found between SPONCS and binocular test performances.

#### Correlations with VF-14

At visit 1, VF-14 scores showed strong correlations with performance of the first operated eye on qCA, Ori, and all qCSF variables except csf1 and csf12, indicating that patients with lower perceived visual function also perform worse on these tests. Some correlations are also observed between VF-14 at visit 1 and performance of the second operated eye on qCSF test. By visit 4, these strong correlations were no longer observed. Also, no significant correlations were found between VF-14 and binocular test performance at either visit 1 or visit 4.

#### Correlations with MoCA

MoCA scores showed strong correlations with high-level visual function tests, such as Ori, CMot, and VSrch, at both visit 1 and visit 4, indicating that higher cognitive function is associated with better performance on complex visual tests. Interestingly, MoCA scores were significantly correlated with lower performance on certain tests performed with the first operated eye, such as qCA, aulcsf, csf12, and csf18. In controls, the strong correlations between MoCA and high-level visual function were not observed, underscoring that patients with low vision may rely more on cognitive resources to compensate for visual deficits.

## Discussion

The primary aim of this work was to study how cataracts and cataract surgery affect various visual abilities. We found that, overall, performance on visual function tests is lower in patients with cataracts compared with healthy controls. Patients were particularly affected in VA and contrast sensitivity, as previously shown (Liu et al., 2017; Shandiz et al., 2011), and they tended to perform worse in Ori and VSrch. Please note that because VSrch was performed binocularly by patients and monocularly by controls, and given the large effect, it is reasonable to assume that a significant difference would have been observed if both populations had performed the test under the same conditions. However, importantly, no visual deficits were found for the CMot. These results suggest that cataracts, which affect the initial stages of visual processing, strongly affect low-level visual processing, but have less influence on higher level processing. In other words, visual deficits to the initial stages of visual processing do not compromise all visual processing, highlighting the complexity of the visual system. The complexity of the visual system is further reflected in the weak correlations between performances in different tests (Supplementary Figure S5), replicating findings from previous research (Bosten, Mollon, Peterzell, & Webster, 2017; Peterzell & Kennedy, 2016; Tulver, 2019). Postoperatively, the major significant improvement occurred for VA, where performance increased after each operation both for binocular and monocular testing. A significant improvement was also detected for contrast sensitivity measures, especially for the first operated eye, and, surprisingly, for CMot after operation on the first eye. CMot was also the only test showing a significant performance increase one month after the last operation (i.e., between visit 3 and visit 4). Performances in Ori showed a tendency of recovery in the binocular testing, whereas cataract surgery did not improve performances in VSrch and RT tests. These findings emphasize the effectiveness of the surgical procedure in agreement with the current literature (Gupta et al., 2017; Qin, Conti, & Singh, 2018), but also show that the benefits are not general, but rather restricted to a subset of visual functions.

The patients included in this study had moderate cataracts in the first operated eye, and similar or even milder cataracts in the second operated eye. Therefore, the greater performance increase observed in contrast sensitivity for the first operated eye compared with the second operated eye is not surprising. Also, contrast sensitivity mainly improved at spatial frequencies corresponding to the peak of human contrast sensitivity (i.e., csf 1.5, 3, 6, and 12; Dorr et al., 2017). Similarly, cataract severity as assessed with SPONCS grading significantly correlated with contrast acuity and contrast sensitivity only for the first operated eye.

The not-so-severe cataract conditions were also reflected in the VF-14 scores, where the mean score was greater than 80 already at visit 1, indicating that patients in Switzerland may undergo surgery quite early in the disease process. In comparison, Alonzo et al. (1997) found that mean preoperative scores in a Danish patients’ population were 76.3 ± 16.8, in a Spanish patient population 63.3 ± 27.0, and in U.S. patients at 75.5 ± 16.6. Therefore, effects sizes might be even greater in cohorts of patients with more advanced cataract stages. Testing more severely affected populations will also clarify whether higher-level visual functions, such as CMot, are at some point also affected by the poor visual input caused by cataracts. It is interesting to note that patients mainly associated VF-14 with impairment in VA and contrast sensitivity of the first operated eye. After surgery, the VF-14 score did not improve significantly. However, there was no significant correlation between VF-14 and tests performances, possibly suggesting that patients still have some discomfort that is not associated with a particular visual function. More research is needed to determine whether the lack of improvement in VF-14 reflects the visual abilities that did not improve after surgery, or whether patients may need more than 1 month to adapt to their new vision and make the best use of it.

The significant improvement in CMot after the first surgery is at a first glance surprising, as patients performed at the same level as controls at baseline. However, it is important to note that older adults are strongly impaired on this test compared with young adults (Shaqiri et al., 2019), leaving significant space for improvement. One possible explanation is that the observed performance improvement reflects a learning effect. Alternatively, it may be that patients, when they cannot rely on clear lenses, learn to solve the test by other cues, which leads to the strong correlations with the MoCA score. After surgery, the patients can suddenly see the stimuli more clearly, and they have the learning, resulting in the observed improvement. Better cognitive scores also significantly correlated with better performance on the Ori, VSrch, and RT tests. This finding emphasizes the importance of cognitive resources in performing complex visual tasks, especially when low-level vision is unreliable, as highlighted by the absence of correlations between vision and MoCA in controls and by the negative correlation between the MoCA, qCA, and qCSF at visit 1 in patients. This finding is consistent with the literature suggesting that high cognitive performance supports better visual processing, particularly in older adults (Garobbio et al., 2022; Vu et al., 2021). Because there was no significant difference in patients vs. controls regarding the MoCA, and no significant changes were seen in the MoCA scores of patients between visit 1 and visit 4, it seems that cognitive function is not affected by changes in vision, but rather that cognitive function can condition visual performance. However, there is also evidence that improved visual performance after cataract surgery could potentially have a positive impact on cognitive function. For example, ACT (Adult Changes in Thought) showed that patients who underwent cataract surgery had an up to a 30% lower risk of developing dementia compared with those who had cataracts but did not have surgery (Lee et al., 2022). This has been linked to improved visual perception after surgery, although the exact mechanisms are not yet fully understood. It is hypothesized that the improved sensory perception reduces cognitive load and could, therefore, support cognitive function (Lee et al., 2022). Another study suggests that people benefit from improved quality of life, mental health, and possibly better cognitive performance after cataract surgery, because visual clarity may have direct and indirect effects on the brain. Especially in older people, improved visual performance can promote social interaction and mental activity, both of which are important factors for cognitive health (Ishii, Kabata, & Oshika, 2008). This finding highlights a key consideration for clinical decision-making: assessing a patient's cognitive function can help to predict their performance on higher-level visual tasks and determine whether cataract surgery might also improve cognitive abilities and help maintain independence. However, further studies using better cognitive assessment are needed to clarify the cause-effect direction of the observed correlations between visual and cognitive domains.

Performance in CMot was the only one to increase one month after the last surgery (i.e., between visit 3 and visit 4). Because performance on the other visual functions did not change, we can assume that complete recovery of postoperative corneal edema, which could impact visual function, was already present at visit 3. Therefore, the improvement in CMot may be attributed to learning effects, cognitive compensation, or possibly also to brain rewiring.

Our study has several limitations. Power analysis shows that an effect size of at least 0.78 would be required to achieve statistical significance with a sample size of 28 and a power of 80%. Thus, studies with larger sample sizes are needed to confirm the observed trends and explore the mechanisms underlying the interaction between cognitive and visual functions. Testing a population with more severe cataracts (VA closer to legal blindness) might strengthen the results. Finally, the lack of robust correlations between the VF-14 and binocular performance as well as monocular 1 postoperatively warrants further exploration to improve subjective tools for assessing functional vision.

## Conclusions

Overall, cataract surgery seems to be optimized to increase VA of the first operated eye, even though other visual functions can also benefit from it. It remains unclear whether the improvement in the tests other than VA reflect the role of VA in performing these tests or whether they take benefit otherwise. These findings emphasize the importance of considering a wide range of visual abilities in managing patients with visual impairment, as their daily life vision depends on all of them. High-level visual processing in patients are influenced not only by visual, but also by cognitive aspects, highlighting the importance of cognitive training in managing visual diseases.

## Supplementary Material

Supplement 1
