# Supplementary material for: To what extent do cataracts and cataract surgery change perception?
Source: J Vis. 2025 Aug 27;25(10):13. doi: 10.1167/jov.25.10.13 (PMC12395818; doi:10.1167/jov.25.10.13)
Supplement: Supplement 1 [file jovi-25-10-13_s001.pdf]

Supplementary Material for

To what extent do cataracts and cataract surgery change perception?

Authors: Simona Garobbio[a*1], Hanna Zuche[b,1], Ursula Hall[b,c], Nina L. Giudici[b], Chrysoula Gabrani[b,c], Hendrik P.N. Scholl[d,e,f], and Michael H. Herzog[a]

[1] SG and HZ contributed equally to the study

Affiliations:

[a] Laboratory of Psychophysics, Brain Mind Institute, École Polytechnique Fédérale de Lausanne (EPFL), Lausanne, Switzerland

[b] Department of Ophthalmology, University Hospital Basel, Basel, Switzerland

[c] Institute of Molecular and Clinical Ophthalmology Basel, Basel, Switzerland

[d] Department of Clinical Pharmacology, Medical University of Vienna, Vienna, Austria

[e] Pallas Kliniken AG, Pallas Klinik Zürich, Zürich, Switzerland

[f] European Vision Institute, Basel, Switzerland

[*]**Corresponding author**: Simona Garobbio, Laboratory of Psychophysics, Brain Mind Institute, School of Life Sciences, EFPL, CH-1015 Lausanne, Switzerland

Phone number: +41216937228

Email: simona.garobbio@epfl.ch

Keywords: cataracts, cataract surgery; visual function tests, vision vs. cognition

Table S1 – Number of outliers excluded based on test-retest

Binocular					1st operated eye	2nd operated eye
VA	CMot	Ori	VSrch	RT	Ori	CMot
2	3	2	6	8	2	0

Acronyms: VA = Freiburg visual acuity; CMot = coherent motion; Ori= orientation discrimination; VSrch = visual search, RT = reaction time

Table S2 – Test-retest reliability of visual functional tests

Variable	Statistics				
	ICC31	*F*	*df1*	*df2*	*p*
**Binocular**					
VA	0.88	15.225	109	109	9.74e-37
CMot	0.65	4.71	99	99	9.86e-14
Ori	0.79	8.46	99	99	6.26e-23
VSrch	0.95	39.323	105	105	2.27e-55
RT	0.86	12.809	103	103	1.85e-31
**1st operated eye**					
Ori	0.78	8.278	94	94	1.64e-21
**2nd operated eye**					
CMot_2OE	0.76	7.391	107	107	3.45e-22

Intraclass correlation of type (3,1) were computed for each visual test. ICCs for VSrch showed excellent reliability (i.e., ICCs > 0.90; Koo & Li, 2016), while all others ICCs indicated good reliability (i.e., ICCs between 0.75 and 0.90), except for CMot which showed moderate reliability (i.e., ICCs between 0.50 and 0.75). Importantly, no one showed poor reliability (i.e., ICCs < 0.50).

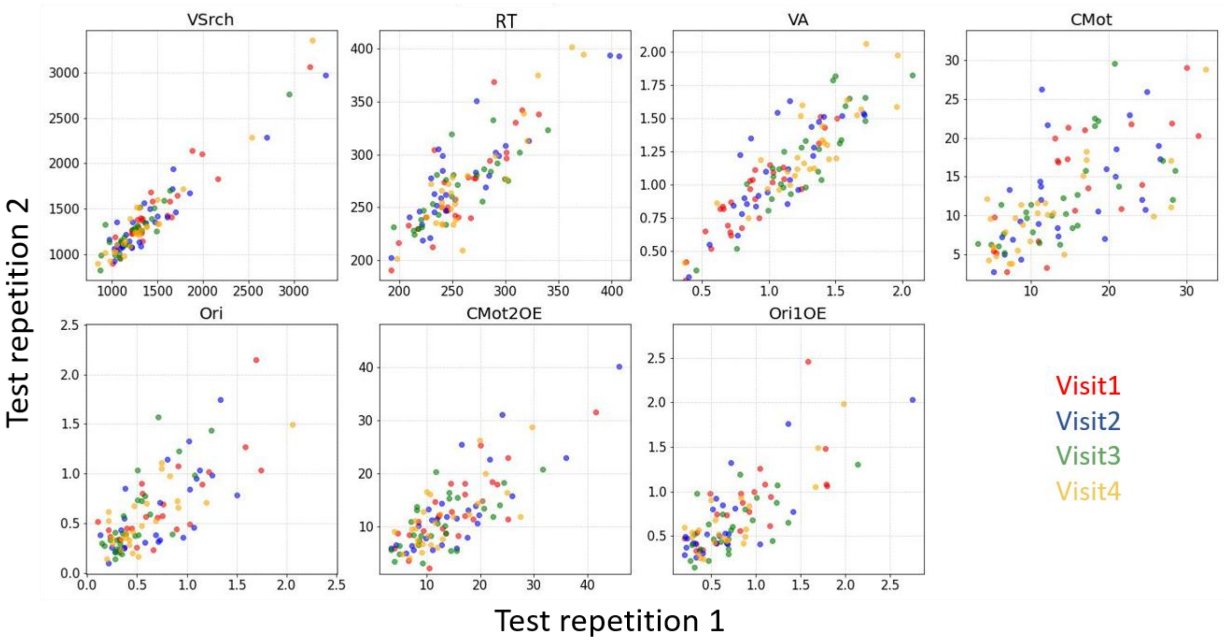

Figure S1: Scatter plots were generated to visualize test-retest reliability. Points along the diagonal indicate that participants achieved the same score in the two test repetitions. In all scatterplots, points clustered around the diagonal, suggesting stable test-retest performance. Red dots represent scores at Visit1, blue dots represent scores at Visit2, green dots represent scores at Visit3, and yellow dots represent scores at Visit4. CMot2OE is used to denote coherent motion test performed with the 2nd operated eye; and Ori1OE corresponds to orientation discrimination test performed with the 1st operated eye.

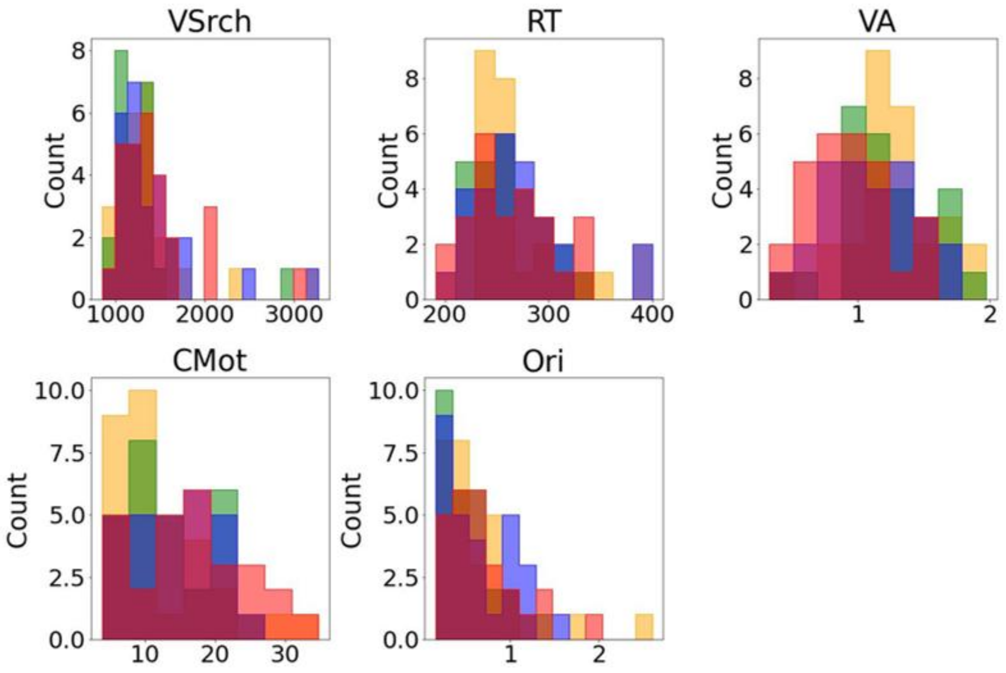

Figure S2: Frequency histograms of the five functional variables extracted from the functional tests performed binocularly. Red represents the score distribution at Visit1, blue at Visit2, green at Visit3, and yellow at Visit4.

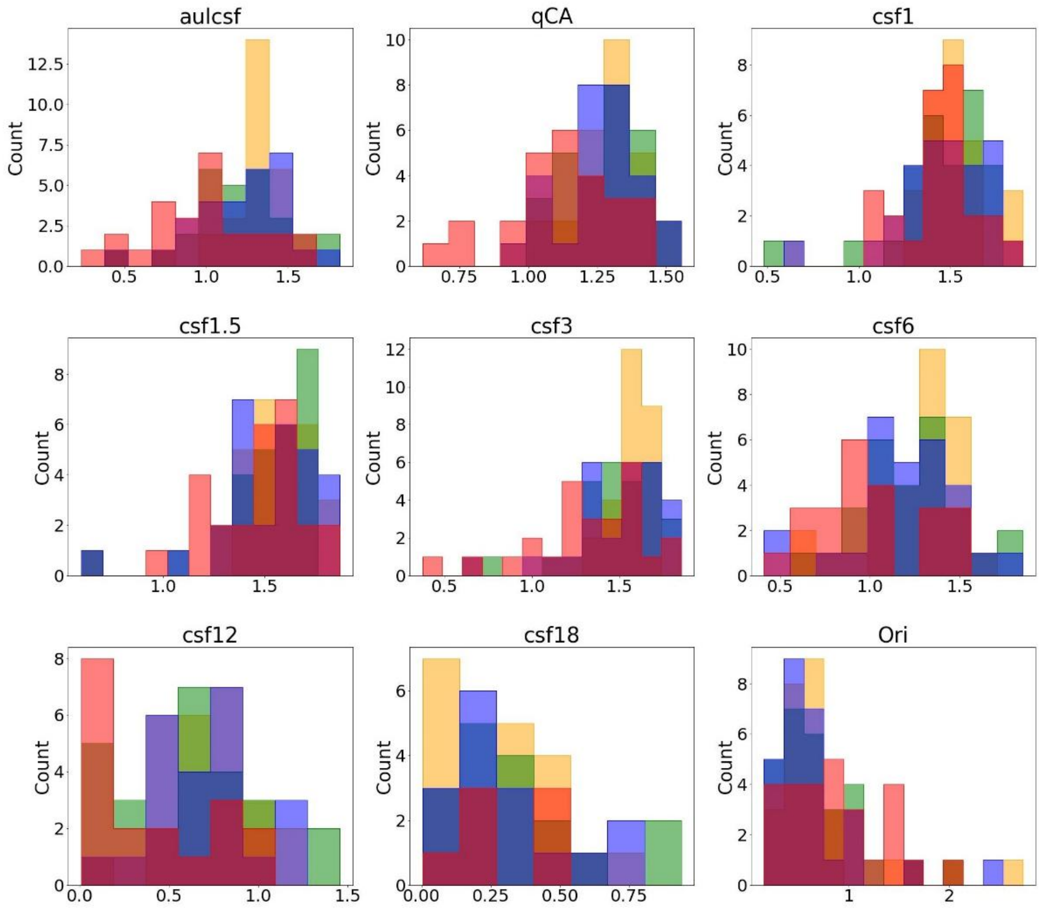

Figure S3: Frequency histograms of the nine functional variables extracted from the functional tests performed monocularly with the first operated eye. New acronyms: aulcsf = area underneath the logarithmis contrast sensitivity function, qCA = contrast acuity, csf1 – csf18 = contrast sensitivity values of the spatial frequencies at 1 – 18 CPDs, respectively. Red represents the score distribution at Visit1, blue at Visit2, green at Visit3, and yellow at Visit4.

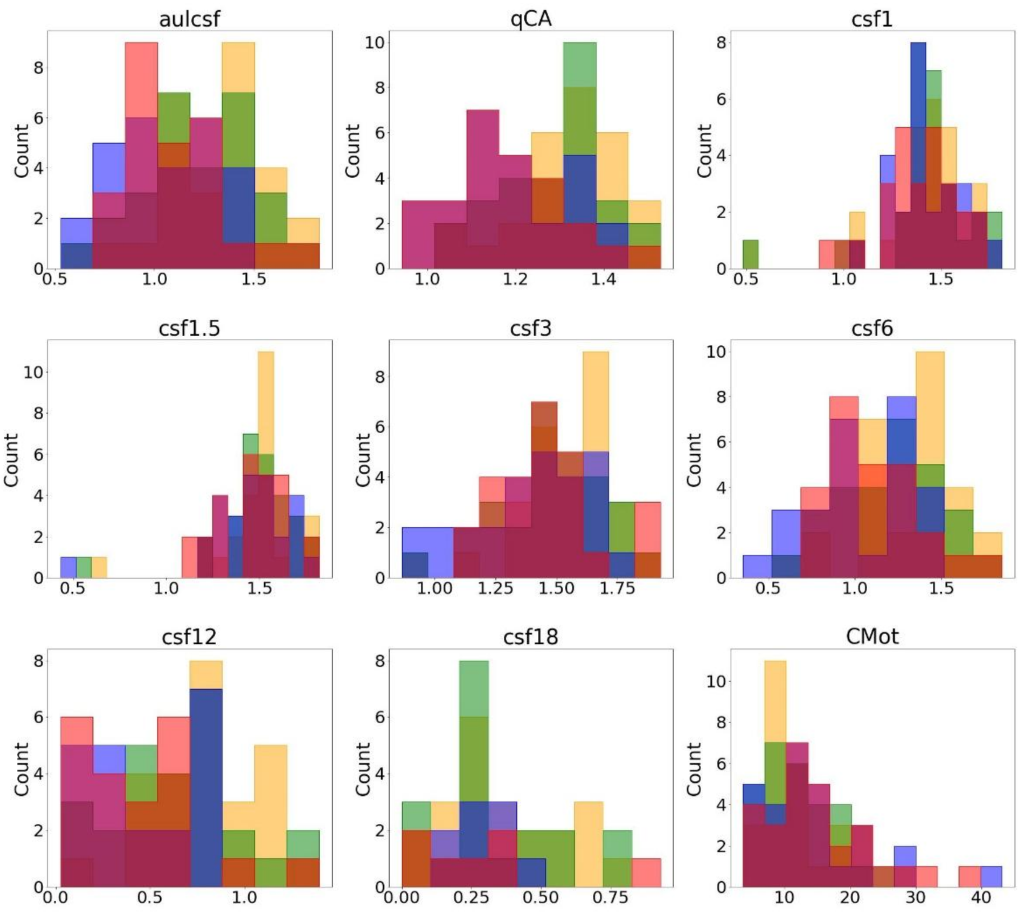

Figure S4: Frequency histograms of the nine functional variables extracted from the functional tests performed monocularly with the second operated eye. Red represents the score distribution at Visit1, blue at Visit2, green at Visit3, and yellow at Visit4.

Table S3 – Comparison of group demographics for patients and controls

	Patients	Controls
N	28	28
Gender (F/M)	11/17	18/10
Age[a]	72.29 ± 7.62	68.36 ± 7.23
BMI	25.45 ± 3.62	24.34 ± 3.20
Smoking years	19.43 ± 21.89	7.0 ±12.27
MoCa	25.46 ± 2.91	26.68 ± 2.30

[a]Age was not significantly different between the two groups (t(53.85)=-1.98, *p*=0.053).

Table S4 – Number of non-missing values, mean, and standard error for each test at each visit

	Visit1			Visit2			Visit3			Visit4		
	n	*M*	*SE*	*n*	*M*	*SE*	*n*	*M*	*SE*	*n*	*M*	*SE*
Binocular												
**VA**	28	0.906	0.299	27	1.090	0.323	27	1.238	0.345	28	1.279	0.342
**Ori**	26	0.710	0.402	28	0.642	0.368	28	0.538	0.312	28	0.635	0.511
**CMot**	27	16.788	7.820	27	14.377	6.086	27	13.151	5.888	28	11.991	7.226
**RT**	25	263.16	39.16	27	269.42	47.46	26	261.69	31.79	26	269.85	46.62
**VSrch**	27	1448.1	443.4	27	1413.2	475.1	26	1287.0	373.7	26	1357.5	495.38
1st operated eye												
**aulcsf**	26	0.969	0.341	28	1.215	0.289	28	1.226	0.283	28	1.278	0.230
**qCA**	26	1.116	0.207	28	1.258	0.141	28	1.265	0.136	28	1.285	0.128
**csf1**	26	1.430	0.202	28	1.458	0.252	28	1.457	0.267	28	1.492	0.227
**csf1.5**	26	1.465	0.209	28	1.509	0.252	28	1.515	0.243	28	1.553	0.222
**csf3**	26	1.317	0.331	28	1.490	0.262	28	1.505	0.228	28	1.550	0.210
**csf6**	23	0.993	0.308	28	1.210	0.299	28	1.220	0.289	28	1.279	0.226
**csf12**	18	0.399	0.347	23	0.697	0.277	26	0.616	0.380	25	0.718	0.226
**csf18**	7	0.305	0.179	16	0.306	0.223	17	0.338	0.257	21	0.270	0.209
**Ori**	27	0.875	0.473	27	0.668	0.454	28	0.717	0.424	27	0.757	0.557
2nd operated eye												
**aulcsf**	26	1.107	0.252	27	1.049	0.266	28	1.215	0.271	28	1.305	0.268
**qCA**	26	1.182	0.129	27	1.183	0.133	28	1.288	0.125	28	1.317	0.116
**csf1**	26	1.381	0.194	26	1.427	0.167	28	1.412	0.251	28	1.428	0.246
**csf1.5**	26	1.459	0.178	27	1.433	0.253	28	1.476	0.241	28	1.494	0.223
**csf3**	26	1.459	0.205	27	1.389	0.239	28	1.478	0.221	28	1.534	0.213
**csf6**	26	1.113	0.276	27	1.022	0.302	28	1.207	0.286	28	1.316	0.285
**csf12**	21	0.451	0.344	21	0.441	0.282	26	0.657	0.329	26	0.789	0.274
**csf18**	7	0.300	0.311	9	0.262	0.093	19	0.331	0.227	22	0.362	0.222
**CMot**	28	15.258	7.809	28	14.561	8.454	28	11.865	5.505	28	12.465	5.941

A higher score indicates better performance in all tests, except for Ori, CMot, RT, and VSrch where lower scores indicate better performance.

Table S5 – Mean and standard error of the functional variables for the control group

	Controls		
	n	M	SE
Non-dominant eye			
**VA**	28	1.165	0.359
**Ori**	28	0.552	0.237
**CMot**	27	15.135	7.195
**VSrch**	28	1238.087	234.328
**aulcsf**	28	1.461	0.186
**qCA**	28	1.381	0.099
**csf1**	28	1.599	0.152
**csf1.5**	28	1.677	0.136

**csf3**	28	1.692	0.132
**csf6**	28	1.438	0.187
**csf12**	28	0.866	0.258
**csf18**	28	0.400	0.282

A higher score indicates better performance in all tests, except for Ori, CMot, and VSrch. The scores are on the same scale as those for patients in Table S4, as both controls and patients performed the same tests with the same setup.

Table S6 – Results from Mann-Whitney U test comparing test performances between patients and controls

**Variable**	**Statistics**				
	U	***p***	***p-BH***	***RBC***	***d***
Patients binocular vs. non-dominant eye controls					
**VA**	546.0	0.012*	0.048*	-0.393	-0.855
**Ori**	291.0	0.209	0.418	0.201	0.410
**CMot**	314.0	0.387	0.418	0.139	0.281
**VSrch**	270.0	0.070	0.210	0.286	0.597
Patients 1st operated eye vs. non-dominant eye controls					
**aulcsf**	650.0	7.7e-7*	7.0e-6*	-0.786	-2.543
**qCA**	641.5	1.6e-5*	1.3e-5*	-0.762	-2.353
**csf1**	546.5	0.002*	0.005*	-0.501	-1.158
**csf1.5**	599.0	4.9e-5*	2.5e-4*	-0.646	-1.693
**csf3**	640.5	1.8e-6*	1.3e-5*	-0.760	-2.339
**csf6**	569.0	3.1e-6*	1.8e-5*	-0.767	-2.391
**csf12**	419.5	1.7e-4*	6.8e-4*	-0.665	-1.781
**csf18**	116.0	0.470	0.470	-0.184	-0.374
**Ori**	218.0	0.007*	0.014*	0.423	0.934
Patients 2nd operated eye vs. non-dominant eye controls					
**aulcsf**	640.5	1.8e-6*	1.4e-5*	-0.760	-2.339
**qCA**	647.5	9.6e-7*	9.0e-6*	-0.779	-2.485
**csf1**	586.5	1.2e-4*	3.6e-4*	-0.611	-1.544
**csf1.5**	618.0	1.1e-5*	7.4e-5*	-0.698	-1.949
**csf3**	613.5	1.6e-5*	8.1e-5*	-0.685	-1.880
**csf6**	619.0	1.1e-5*	7.4e-5*	-0.701	-1.966
**csf12**	478.5	3.5e-5*	1.4e-4*	-0.709	-2.011
**csf18**	122.0	0.332	0.664	-0.245	-0.505
**CMot**	377.0	0.993	0.993	0.003	0.006

Bonferroni-Holm correction for multiple comparisons was applied across the tests performed binocularly, with the 1st operated eye, and with the 2nd operated eye of patients. An asterisk (*) indicates a significant difference between the two groups ($p < 0.05$). Rank-biserial correlation (RBC) was converted to Cohen's *d* using the formula: Cohen's d equals twice the RBC divided by the square root of one minus RBC squared. A higher score indicates better performance in all tests, except for Ori, CMot, and VSrch. Therefore, a negative effect size indicates better performance for controls compared to patients in all tests except for Ori, CMot, and VSrch, where a positive effect size indicates better performance for controls.

Table S7 – Statistical results from linear mixed-effect models (LMM)

Variable		Statistics				
		Coefficient	*Standard error*	*z-value*	*p-value*	*CI [0.025 0.975]*
Binocular						
**VA**	Intercept<br>Visit<br>Subj. intercept var	0.817<br>0.125<br>0.068	0.068<br>0.017<br>0.124	12.071<br>7.424	1.5e-33<br>1.1e-13	[0.684 0.949]<br>[0.092 0.158]
**Ori**	Intercept<br>Visit<br>Subj. intercept var	0.746<br>-0.043<br>0.094	0.087<br>0.024<br>0.132	8.527<br>-1.801	1.5e-17<br>0.072	[0.574 0.917]<br>[-0.089 0.004]
**CMot**	Intercept<br>Visit<br>Subj. intercept var	18.125<br>-1.566<br>36.989	1.358<br>0.262<br>4.053	13.349<br>-5.973	1.19e-40<br>2.32e-9	[15.464 20.786]<br>[-2.080 -1.052]
**RT**	Intercept<br>Visit<br>Subj. intercept var	265.796<br>1.349<br>1600.51	8.823<br>1.659<br>29.626	30.125<br>0.813	2.3e-199<br>0.416	[248.50 283.09]<br>[-1.902 4.601]
**VSrch**	Intercept<br>Visit<br>Subj. intercept var	1488.40<br>-23.28<br>226’725	101.121<br>16.971<br>396.872	14.719<br>-1.372	4.9e-49<br>0.170	[1290.2 1686.6]<br>[-56.54 9.982]
1st operated eye						
**qCA**	Intercept<br>Visit<br>Subj. intercept var	1.104<br>0.051<br>0.009	0.035<br>0.011<br>0.033	31.690<br>4.747	2.1e-220<br>2.1e-6	[1.036 1.172]<br>[0.030 0.072]
**Ori**	Intercept<br>Visit<br>Subj. intercept var	0.860<br>-0.039<br>0.152	0.103<br>0.026<br>0.186	8.390<br>-1.483	4.9e-17<br>0.138	[0.659 1.061]<br>[-0.090 0.012]
**aulcsf**	Intercept<br>Visit<br>Subj. intercept var	0.944<br>0.092<br>0.044	0.062<br>0.017<br>0.084	15.141<br>5.251	8.7e-52<br>1.5e-7	[0.822 1.067]<br>[0.057 0.126]
**csf1**	Intercept<br>Visit<br>Subj. intercept var	1.411<br>0.019<br>0.031	0.050<br>0.014<br>0.073	27.948<br>1.386	6.9e-171<br>0.166	[1.312 1.510]<br>[-0.008 0.046]
**csf1.5**	Intercept<br>Visit<br>Subj. intercept var	1.443<br>0.027<br>0.032	0.048<br>0.013<br>0.081	29.779<br>2-169	7.4e-195<br>0.030	[1.348 1.538]<br>[0.003 0.052]
**csf3**	Intercept<br>Visit<br>Subj. intercept var	1.293<br>0.070<br>0.037	0.056<br>0.015<br>0.079	23.235<br>4.557	2.0e-119<br>5.2e-6	[1.184 1.402]<br>[0.040 0.099]
**csf6**	Intercept<br>Visit<br>Subj. intercept var	0.979<br>0.081<br>0.042	0.062<br>0.017<br>0.083	15.773<br>4.662	4.8e-56<br>3.1e-6	[0.857 1.100]<br>[0.047 0.115]
**csf12**	Intercept<br>Visit<br>Subj. intercept var	0.381<br>0.085<br>0.039	0.081<br>0.025<br>0.079	4.685<br>3.400	2.8e-6<br>0.001	[0.221 0.540]<br>[0.036 0.134]
**csf18**	Intercept<br>Visit<br>Subj. intercept var	0.304<br>-0.006<br>0.024	0.073<br>0.021<br>0.079	4.179<br>-0.293	2.9e-5<br>0.770	[0.162 0.447]<br>[-0.047 0.035]
**2nd operated eye**						
**qCA**	Intercept<br>Visit<br>Subj. intercept var	1.113<br>0.052<br>0.006	0.028<br>0.009<br>0.029	39.665<br>6.099	0.00<br>1.1e-9	[1.058 1.168]<br>[0.035 0.069]
**CMot**	Intercept<br>Visit<br>Subj. intercept var	16.306<br>-1.107<br>34.644	1.430<br>0.328<br>3.101	11.404<br>-3.376	3.9e-30<br>0.001	[13.503 19.108]<br>[-1.750 -0.464]
**aulcsf**	Intercept	0.976	0.058	16.904	4.23e-64	[0.863 1.089]

	Visit<br>Subj. intercept var	0.078<br>0.038	0.016<br>0.079	4.837	1.32e-6	[0.046 0.109]
**csf1**	Intercept<br>Visit<br>Subj. intercept var	1.364<br>0.017<br>0.024	0.049<br>0.014<br>0.063	28.053<br>1.189	3.6e-173<br>0.234	[1.269 1.459]<br>[-0.011 0.044]
**csf1.5**	Intercept<br>Visit<br>Subj. intercept var	1.424<br>0.016<br>0.031	0.047<br>0.012<br>0.080	30.014<br>1.341	6.4e-198<br>0.180	[1.331 1.517]<br>[-0.008 0.040]
**csf3**	Intercept<br>Visit<br>Subj. intercept var	1.383<br>0.033<br>0.027	0.048<br>0.013<br>0.069	29.086<br>2.520	5.3e-186<br>0.012	[1.290 1.477]<br>[0.007 0.058]
**csf6**	Intercept<br>Visit<br>Subj. intercept var	0.963<br>0.081<br>0.038	0.064<br>0.019<br>0.073	14.998<br>4.264	7.6e-51<br>2.0e-5	[0.837 1.089]<br>[0.044 0.118]
**csf12**	Intercept<br>Visit<br>Subj. intercept var	0.263<br>0.126<br>0.039	0.075<br>0.023<br>0.077	3.515<br>5.577	4.4e-4<br>2.4e-8	[0.116 0.410]<br>[0.082 0.171]
**csf18**	Intercept<br>Visit<br>Subj. intercept var	0.212<br>0.037<br>0.012	0.090<br>0.027<br>0.055	2.359<br>1.372	0.018<br>0.170	[0.036 0.388]<br>[0.016 0.089]

The Intercept is the average score at baseline. The Visit coefficient indicates the change in the score with each visit. The Subject intercept variance shows how much individual subjects' baseline measurements deviate from the average intercept. A high score indicates better performance in all tests except for Ori, CMot, RT and VSrch. Thus, a positive Visit coefficient indicates performance improvement across visits in all tests except for Ori, CMot, RT and VSrch, where performance improvement across time is indicated by a negative Visit coefficient.

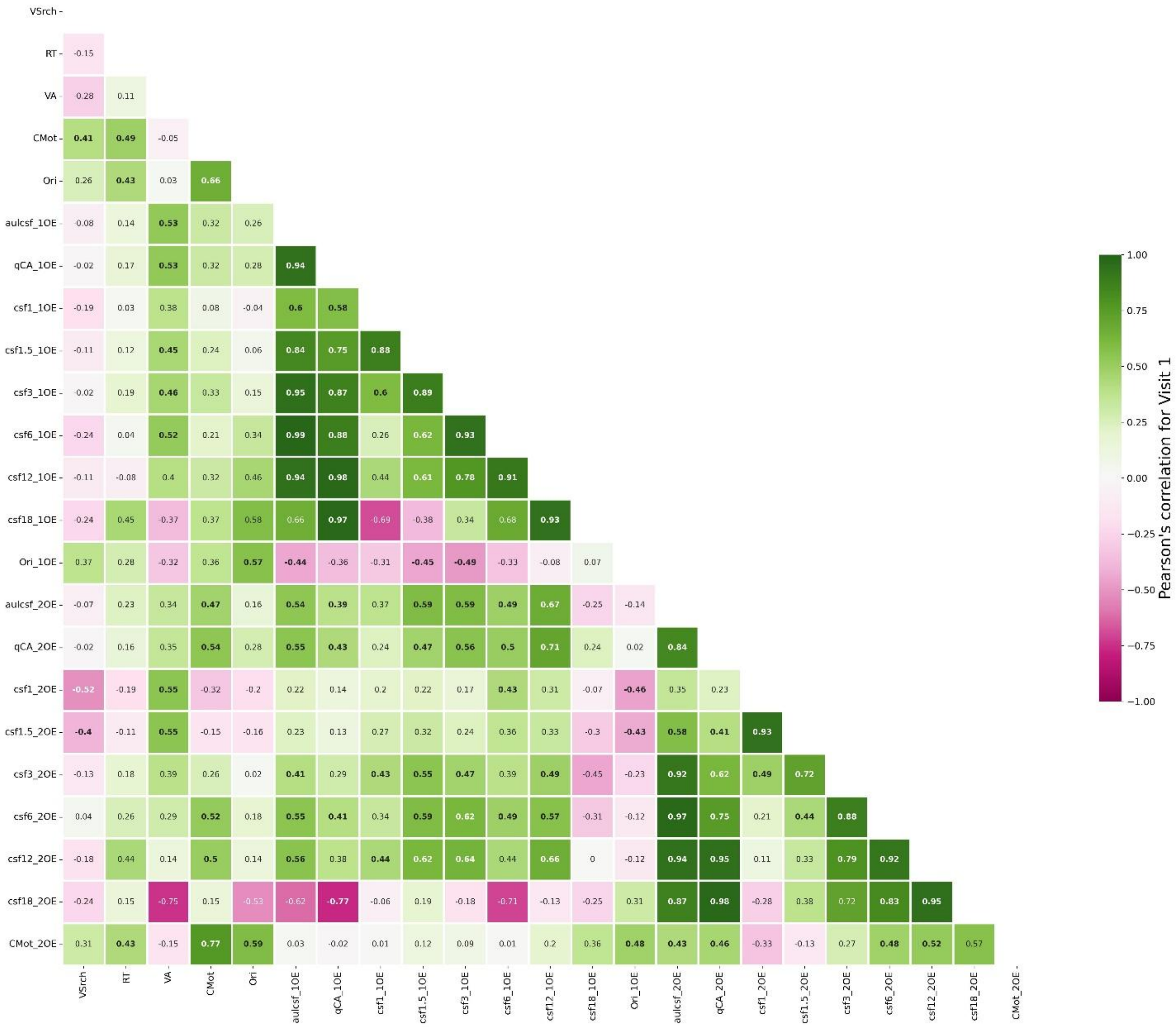

Figure S5: Between-tests Pearson correlation coefficients for Visit 1 including tests performed binocularly (VSrch, RT, VA, CMot, Ori), tests performed with 1st operated eye (aulcsf_1OE, qCA_1OE, csf_1OE, csf1.5_1OE, csf3_1OE, csf6_1OE, csf12_1OE, csf18_1OE, and Ori_1OE), and tests performed with 2nd operated eye (aulcsf_2OE, qCA_2OE, csf_2OE, csf1.5_2OE, csf3_2OE, csf6_2OE, csf12_2OE, csf18_2OE, and CMot_2OE). Bold numbers indicate significant results ($p < 0.05$, we did not correct for multiple comparisons). The color scale ranging from pink to green represents effect sizes from $r = -1$ to $r = 1$ (white corresponds to $r = 0$). Please note that pairwise deletion was used to compute correlations, and that better performance is indicated by an higher score in all tests except for VSrch, RT, CMot, and Ori.

In general, correlations are weak to moderate, with quartiles at 0.02, 0.34, and 0.57. Binocular tests mainly show weak correlations among themselves, with some exceptions for the coherent motion test. Also, weak correlations are found between the binocular and monocular tests, where

only, as expected, VA correlates with qCA and the low spatial frequencies of the qCSF test. Correlations between the same test performed on the first and second operated eyes are of moderate strength, indicating a degree of interocular variability. Strong correlations are primarily observed between different spatial frequencies within the same eye (e.g., csf1.5_1OE, csf3_1OE, etc.), reflecting internal consistency within the qCSF test. However, not all CSF frequencies correlate strongly, even within the same eye (e.g., low vs. higher spatial frequencies as csf1 which only weakly correlate with csf6, csf12, and csf18 for both eyes).

Overall, our results extend previous findings by demonstrating that visual performance is multifactorial not only in healthy adults but also in older adults with cataract. In other words, performance in one visual test is not necessarily indicative of performance in another. Please note that the current sample size is small, and the results should be interpreted with caution.
